# Sickness absence patterns and trends in the health care sector: 5-year monitoring of female municipal employees in the health and care sectors in Norway and Denmark

**DOI:** 10.1186/1478-4491-12-37

**Published:** 2014-07-08

**Authors:** Line Krane, Roar Johnsen, Nils Fleten, Claus Vinther Nielsen, Christina M Stapelfeldt, Chris Jensen, Tonje Braaten

**Affiliations:** 1 Department of Community Medicine, Faculty of Health Sciences, UiT The Arctic University of Norway, NO-9037 Tromsø, Norway; 2 Department of Public Health and General Practice, Faculty of Medicine, Norwegian University of Science and Technology, Trondheim NO-7491, Norway; 3 Section of Social Medicine and Rehabilitation, Department of Public Health, Aarhus University, Nordre Ringgade 1, Aarhus C DK-8000, Denmark; 4 Clinical Social Medicine and Rehabilitation, Marselisborg Center, Public Health and Quality Improvement, Central Denmark Region, P.P. Ørums Gade 11, Aarhus C DK-8000, Denmark; 5 National Center for Occupational Rehabilitation, Rauland NO-3864, Norway

**Keywords:** Comparative study, Denmark, Health and care units, Longitudinal study, Norway, Sick leave pattern

## Abstract

**Background:**

Sickness absence is a growing public health problem in Norway and Denmark, with the highest absence rates being registered in Norway. We compared time trends in sickness absence patterns of municipal employees in the health and care sectors in Norway and Denmark.

**Methods:**

Data from 2004 to 2008 were extracted from the personnel registers of the municipalities of Kristiansand, Norway, and Aarhus, Denmark, for 3,181 and 8,545 female employees, respectively. Age-specific comparative statistics on sickness absence rates (number of calendar days of sickness absence/possible working days) and number of sick leave episodes were calculated for each year of the study period.

**Results:**

There was an overall increasing trend in sickness absence rates in Denmark (*P* = 0.002), where rates were highest in the 20–29- (*P* = 0.01) and 50–59-year-old age groups (*P* = 0.03). Sickness absence rates in Norway were stable, except for an increase in the 20–29-year-old age group (*P* = 0.004). In both Norway and Denmark, the mean number of sick leave episodes increased (*P* <0.0001 and *P* <0.0001, respectively) in all age groups except for the 30–39- and 60–67-year-old age groups. The proportion of employees without sickness absence was higher in Norway than in Denmark. Both short-term and long-term absence increased in Denmark (*P* = 0.003 and *P* <0.0001, respectively), while in Norway, only short-term absence increased (*P* = 0.09).

**Conclusions:**

We found an overall increase in sickness absence rates in Denmark, while the largest overall increase in sick leave episodes was found in Norway. In both countries, the largest increases were observed among young employees. The results indicate that the two countries are converging in regard to sickness absence measured as rates and episodes.

## Background

Sickness absence has become a growing public health challenge in Western societies over the last decades [1-3]. In both Norway and Denmark, the highest sickness absence rates are found in the health and care sectors [4-6]. The economic burden of sickness absence is considerable, and authorities in both countries want to reduce these costs.

Some of the first comparable statistics for sickness absence rates in Norway and Denmark were recorded in 1987, and data from this time and thereafter consistently showed higher rates in Norway than in Denmark [7-9]. The trend in sickness absence from 1987 to 2009 showed a relatively large variation in sickness absence rates in Norway (from approximately 2.5% to 4%) compared to Denmark, where sickness absence rates were far more stable (from approximately 1.6% to 1.9%) [7,9,10]. These numbers were taken from the Labour Force Surveys and indicate the level of sickness absence measured as a rate [7,11]. Currently, the sickness absence rate in Norway is among the highest in Northern Europe, whereas Denmark has far lower rates [12]. However, the sickness absence rates during the last 5 years have decreased slightly in all age groups in Norway, and have increased in all age groups in Denmark [13,14]. Although sickness absence patterns among employees in the health and care sectors have been explored only to a limited extent, previous international studies have indicated increasing rates with age [15].

Sickness benefit policies are important factors in explaining sickness absence patterns. A comparison of these policies in Norway and Denmark might contribute to a better understanding of the underlying causes of sickness absence in the two countries [1,8,11]. Unfortunately, we have not found any comparative studies of sickness absence in the health and care sector from other countries.

Sick leave regulations in Norway and Denmark, which apply to all employees, share some common features: the initial compensation rate is 100%, and there is no waiting period. The employer finances the compensation during the first 16 days of sick leave in Norway, and for the first 14 days, which was extended to 21 days in 2008, in Denmark. After the employer compensation period expires, compensation is fully or partly paid by the public authorities [16-18]. The maximum duration one can receive compensation while on sick leave is 1 year in both countries. It is possible to extend this duration in Denmark if the relevant authorities or a physician require on-going evaluations of work capacity and if the employee is awaiting medical treatment, has a work injury claim in progress, or has a deadly disease. The weekly maximum disbursements for sick leave compensation are higher in Norway than in Denmark [8,16]. However, as all government employees, those in the health and care sector in Denmark receive full pay during sick leave, and thus have no more economic incentive to reduce their absenteeism than their counterparts in Norway. Regulations concerning job security are different in Norway and Denmark [8,18,19]; employees in Denmark might lose their job while on sick leave. Sickness absence tends to be negatively correlated with unemployment, and the unemployment level in Denmark has been higher than that in Norway for several years [20-22].

Previous studies have shown variation in sickness absence by age [7,23-25]. According to the European Working Conditions Observatory [13], the total level of sickness absence decreased between 2003 and 2008 in Norway in all age groups. The same observatory [14] reported a slightly increasing trend of sickness absence between 2003 and 2008 in Denmark. It has been reported that older employees tend to have more sickness absence than younger employees [11], and larger age differences in sickness absence rates have been shown in Norway than in Denmark [7]. In Norway, the sickness absence rates increased with age, while in Denmark, the rates decreased in the 60–67-year-old age group compared to the 50–59-year-old age group [7]. Moreover, the mean number and frequency of sick leave episodes slightly increased in Norway between 1975 and 2002 [26]. Comparisons between countries may be impeded by differences in the size of a given industry, and the sex, age, etc., of its employees [15]. Strict comparisons cannot be made unless employees are selected from the same industry and have the same type of job. Therefore, we have chosen to study the health and care sectors in Norway and Denmark as we consider the work tasks to be comparable across countries. The municipal health and care sectors include, for example, nursing homes, home care services, and day centers. This study is a part of a larger study where background variables, such as occupation, age, and percentage of employment, were investigated [25]. In this study, we focus on sickness absences patterns and trends over a 5-year period, overall and by age group.

The aim of this comparative study was to assess the development in sickness absence rates, short-term and long-term absence, and frequency of sick leave episodes from 2004 to 2008 in the health and care sectors in the cities of Kristiansand, Norway, and Aarhus, Denmark.

## Methods

### Data

Data recorded in the personnel registers of the municipalities of Kristiansand, Norway, and Aarhus, Denmark, from 2004 to 2008 were used in the present longitudinal cohort study, which included a 5-year follow-up. Sickness absence rates and number of sick leave episodes were calculated for each year of the study period. The sickness absence rate was measured as days of sickness absence as a percentage of possible working days. Short- and long-term absence was calculated on the basis of sickness absence rate.

### Study population

At baseline in 2004, the study population included 2,004 and 4,275 female employees in the municipalities of Kristiansand, Norway, and Aarhus, Denmark, respectively. Including new employees starting their employment during the follow-up period, a total of 3,181 and 8,545 employees, respectively, participated in the study. The increase is due to a natural increase in people in need of care. The study population was restricted to health and care sector employees in order to avoid differences related to job tasks and occupational sectors. Individuals having less than 20% employment were excluded entirely from the study, as were employees on maternity leave, adoption leave, or other kinds of parental leave, and students. The percentage of part-time employment (20–50% of full work hours) is 14.2% in Norway and 0.5% in Denmark [25]. The proportion of male employees in the health and care sector was low in both countries, 10.3% and 4.7%, respectively, too few to achieve satisfactory power, so they were also excluded from the study. The cities were chosen due to a self-sick listed project in Kristiansand and an established collaboration and availability of data in Aarhus, which made these the two most comparable cities [25]*.*

### Measurements

In this study, sick leave patterns were measured in terms of sickness absence rate and number of sick leave episodes [27]. Individual sickness absence rates were calculated as follows:

∑ (calendar days of sickness absence × 5/7) / (∑ (possible working days) × 230(220)/365).

A normal work year is 230 days in Norway and 220 days in Denmark. A single sick leave episode can last from 1 to 365 days. Absences interrupted by 1 day without sick leave compensation, typically on weekends, were registered as a single period. Results presented include sick leave episodes that ended between 2004 and 2008. Absence continuing from one year to another was considered as one consecutive sick leave episode and the actual length was measured. The employees’ age changed successively. The length of sickness absence was categorized for each year as follows: 0 = no absence, >0–10% = absence lasting up to 10%, >10–50% = absence lasting from 10 to 50%, and >50% = absence lasting more than 50% of the working year, which was considered long-term absence. The number of sick leave episodes was categorized as: 0 = no absence, 1 = one sick leave episode, 2–5 = two to five sick leave episodes, and 6–26 = six to 26 sick leave episodes.

### Statistical analyses

Data were analyzed using Stata for Windows, version 12. We performed age-specific comparative descriptive analyses of patterns of sickness absence and sick leave episodes measured by rates and frequencies, with corresponding *P* values for linear trend. Linear and logistic regression models were applied for the trend tests, respectively.

### Approval

The Data Protection Official for Research, through the Norwegian Social Science Data Service, approved the project. The project was subject to the rules for processing personal data, see §7-27 of the Personal Data Regulations. Approval (2012-41-1290) for conducting this register-based study was given by the Danish Data Protecting Agency.

## Results

The sickness absence rate of municipal employees in the health and care sectors of Kristiansand, Norway, and Aarhus, Denmark, changed from 11.9% (95% CI 11.8–12.0) in 2004 to 11.6% (95% CI 11.5–11.7) in 2008 in Kristiansand, compared to 7.1% (95% CI 7.0–7.1) and 8.4% (95% CI 8.4–8.5) in Aarhus in the same years. We observed a significant increasing trend (*P* = 0.002) in the sickness absence rates in Denmark from 2004 to 2008, but not in Norway (Table 1).

**Table 1:** Sickness absence rates by age for municipal employees in the health and care sectors in Kristiansand, Norway, and Aarhus, Denmark, 2004 to 2008

	**N**	**%**	**2004**		**2005**		**2006**		**2007**		**2008**		***P ***^**b**^
**Norway**													
**N**	3,181		2,004		2,052		2,128		2,254		2,375		
			%		%		%		%		%		
**Overall rate with CI**^**a**^			11.9	11.8–12.0	10.4	10.3–10.6	10.4	10.3–10.5	11.6	11.5–11.7	11.6	11.5–11.7	0.43
**Rate per age group (years)**													
20–29	796	25.0	10.6		10.4		9.9		13.0		14.7		0.004
30–39	744	23.4	13.7		11.4		12.4		13.5		12.7		0.63
40–49	813	25.6	10.1		9.7		9.8		11.0		10.0		0.45
50–59	652	20.5	12.4		10.4		10.5		10.9		11.4		0.85
60–67	176	5.5	15.7		11.9		7.8		9.4		10.1		0.13
**Denmark**													
**N**	8,545		4,275		4,691		5,252		5,510		5,315		
			%		%		%		%		%		
**Overall rate with CI**^**a**^			7.1	7.0–7.1	7.1	7.1–7.2	8.3	8.2–8.3	8.8	8.7–8.9	8.4	8.4–8.5	0.002
**Rate per age group (years)**													
20–29	2,123	24.8	6.7		8.5		8.8		10.7		10.3		0.01
30–39	1,790	20.9	8.3		7.6		8.8		9.6		9.8		0.38
40–49	2,098	24.6	6.8		7.0		7.9		8.0		7.4		0.42
50–59	2,084	24.4	7.0		6.8		8.4		8.6		8.1		0.03
60–67	450	5.3	5.8		4.7		6.5		7.4		6.8		0.29

^a^ Between-countries age-adjusted numbers with 95% CIs. ^b^*P* for linear trend. CI, confidence interval.

A significant increase in sickness absence rates was seen in employees aged 20–29 years in both countries (Norway *P* = 0.004, Denmark *P* = 0.01). The increase in this age group was 39% in Norway compared to 54% in Denmark, which also had a 15% increase in the 50–59-year-old age group, with a peak in 2007.

The overall mean number of sick leave episodes in Norway increased significantly during the study period, from 1.8 to 2.3 (Table 2, *P* <0.0001). We also observed a significant increase in sick leave episodes in Denmark, from 2.0 in 2004 to 2.2 in 2008 (Table 2, *P* <0.0001). The increase was only significant in the 20–29- (*P* <0.0001), 40–49- (*P* <0.0001), and 50–59-year-old age groups (*P* = 0.003) in Norway; and in the 20–29- (*P* <0.0001), 40–49- (*P* = 0.01), and 50–59-year-old age gourps (*P* <0.0001) in Denmark. We observed a 50% increase in the number of sick leave episodes in the 20–29-year-old age group in Norway compared to a 20% increase in Denmark.

**Table 2:** Mean number of sick leave episodes by age for municipal employees in health and care sectors in Kristiansand, Norway, and Aarhus, Denmark, 2004 to 2008

	**2004**		**2005**		**2006**		**2007**		**2008**		***P ***^**b**^
**Norway**											
		95% CI		95% CI		95% CI		95% CI		95% CI	
**Overall mean**^**a**^	1.8	1.7–1.9	2.0	1.9–2.0	2.0	1.9–2.1	2.0	2.0–2.1	2.3	2.2–2.4	<0.0001
**Age group (years)**											
20–29	1.6		1.8		2.0		2.2		2.4		<0.0001
30–39	2.1		2.2		2.2		2.0		2.4		0.23
40–49	1.8		2.0		2.1		2.1		2.3		<0.0001
50–59	1.9		2.0		1.9		2.1		2.3		0.003
60–67	1.5		1.5		1.8		1.7		1.8		0.11
**Denmark**											
		95% CI		95% CI		95% CI		95% CI		95% CI	
**Overall mean**^**a**^	2.0	2.0–2.1	2.1	2.0–2.1	2.1	2.1–2.2	2.2	2.2–2.3	2.2	2.2–2.3	<0.0001
**Age group (years)**											
20–29	2.0		2.2		2.1		2.5		2.4		<0.0001
30–39	2.4		2.4		2.3		2.4		2.6		0.09
40–49	2.1		2.2		2.3		2.3		2.3		0.01
50–59	1.8		1.9		2.0		2.0		2.1		<0.0001
60–67	1.4		1.4		1.4		1.4		1.6		0.11

^a^ Between-countries age-adjusted numbers with 95% CIs. ^b^*P* for linear trend. CI, confidence interval.

In Norway, we observed a decreasing proportion of employees without sickness absence (Table 3, *P* = 0.001), whereas the proportion of employees without sickness absence in Denmark remained unchanged between 2004 and 2008 (Table 3). Short-term absence was stable in Norway during the study period, while in Denmark there was a minor decrease from 62.9% in 2004 to 60.1% in 2008 (Table 3, *P* = 0.003).

**Table 3:** Distribution of sickness absence rates according to total duration in proportion of yearly work time, in four categories, for municipal employees in the health and care sectors in Kristiansand, Norway, and Aarhus, Denmark, 2004 to 2008

	**2004**	**2005**	**2006**	**2007**	**2008**	***P ***^**b**^
**Norway**						
**Sickness absence rate**^**a**^						
0%	28.0	28.2	26.3	25.5	24.3	0.001
>0–10%	46.6	46.3	49.3	47.9	47.9	0.09
>10–50%	18.1	18.4	17.3	18.3	20.4	0.08
>50%	8.2	7.0	7.0	8.2	7.4	0.90
**Denmark**						
**Sickness absence rate**^**a**^						
0%	19.9	20.5	20.5	18.9	20.0	0.35
>0–10%	62.9	62.1	60.6	60.9	60.1	0.003
>10–50%	12.6	13.7	13.5	14.1	14.1	0.03
>50%	4.6	3.7	5.3	6.0	5.6	<0.0001

^a^ The numbers are between-countries age adjusted. ^b^*P* for linear trend.

In Norway, the proportion of employees with long-term absence (50% absence or more in a year) showed a fluctuating pattern, decreasing from 8.2% in 2004 to 7.0% in 2005 and 2006, increasing to 8.2% in 2007, and eventually decreasing to 7.4% in 2008. In Denmark, long-term absence followed a significant linear trend (*P* <0.0001), decreasing from 4.6% in 2004, to 3.7% in 2005, increasing again to 5.3% in 2006 and 6.0% in 2007, to finally decrease to 5.6% in 2008.

In Norway, the proportion of employees having 2–5 and 6–26 sick leave episodes increased significantly (Table 4), from 39.8% in 2004 to 42.9% in 2008 (*P* = 0.04) and 5.4% in 2004 to 9.6% in 2008 (*P* <0.0001), respectively. In Denmark, the proportion of employees having 6–26 sick leave episodes increased from 4.5% in 2004 to 7.0% in 2008 (*P* <0.0001), following a significant linear trend (Table 4).

**Table 4:** Distribution of sick leave episodes (sle) per employee in five categories, for municipal employees in the health and care sectors in Kristiansand, Norway, and Aarhus, Denmark, 2004–2008

	**2004**	**2005**	**2006**	**2007**	**2008**	***P ***^**a**^
**Norway**						
**Number of sick leave episodes**						
0 (sle)	31.8	31.5	29.2	28.5	26.9	<0.0001
1(sle)	23.0	20.5	21.1	22.0	20.3	0.18
2–5 (sle)	39.8	41.2	43.2	41.2	42.9	0.04
6–26 (sle)	5.4	6.6	6.3	7.4	9.6	<0.0001
**Denmark**						
**Number of sick leave episodes**						
0 (sle)	20.0	20.5	20.4	19.0	19.5	0.12
1 (sle)	26.1	25.1	24.9	24.5	24.5	0.06
2–5 (sle)	49.2	48.9	49.2	49.7	48.7	0.89
6–26 (sle)	4.5	5.2	5.3	6.7	7.0	<0.0001

^a^*P* for linear trend.

**Figure 1:**
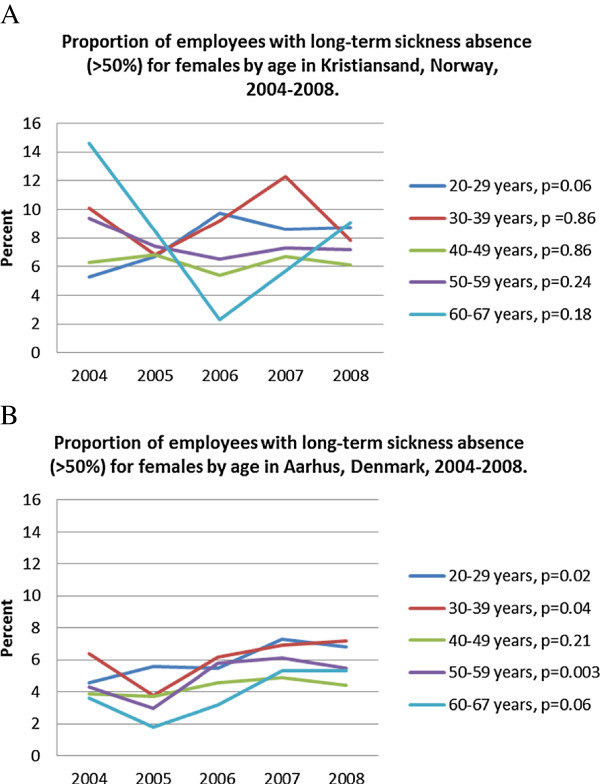
Proportion of employees with long-term sickness absence (>50%) for females by age in Kristiansand, Norway, 2004-2008.

**Figure 2:**
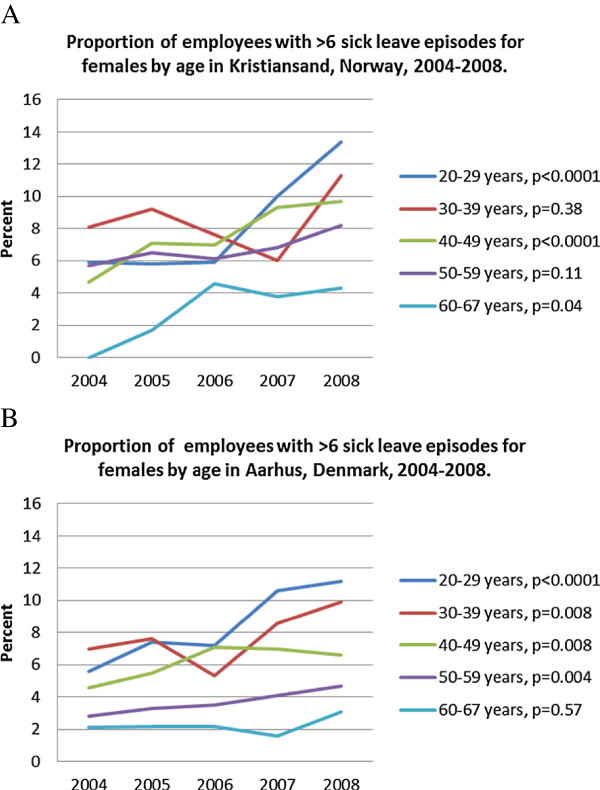
Proportion of employees with long-term sickness absence (>50%) for females by age in Aarhus, Denmark, 2004-2008.

However, in Norway long-term sickness absence increased substantially in the 20–29-year-old age group (Figure 1a, *P* = 0.06), while it decreased in the other age groups. In Denmark, long-term sickness absence increased in all age groups, especially among those aged 20–29 years (Figure 1b, *P* = 0.02).The proportion of employees with more than six sick leave episodes increased in all age-groups in both countries, especially in the 20-29-year age-group (Figures 1a and 2b).

## Discussion

Our results showed that overall sickness absence rates in Kristiansand, Norway, were higher, but stable from 2004 to 2008 compared to Aarhus, Denmark. The overall sickness absence rate in Aarhus, Denmark, remained substantially lower, despite a significant increase during the study period. We found an increasing trend in sickness absence rates in the youngest age groups both in Kristiansand, Norway, and Aarhus, Denmark. Our results are consistent with national sick leave trends for Norway and Denmark, though at higher levels. In Norway, national sick leave rates for female employees in the health and care sectors were stable (10.2% in 2004 and 10.3% in 2008) [5]. In Denmark, national sick leave rates for female employees showed an increase from 3.9% in 2004 to 4.3% in 2008. The increase was even more pronounced among young employees [6]. This is in accordance with our findings.

In our study, the mean number of sick leave episodes increased linearly from 2004 to 2008 in both Norway and Denmark, with the highest increase in Norway. Our results showed a significant, increasing trend of young employees with several sick leave episodes in both Norway and Denmark, and we also found an increase among employees aged 40–59 years in both countries. Although the proportion of employees with sickness absence increased significantly in Norway, it remained significantly lower than in Denmark. We observed a slight increase in short-term absences in Norway, but a decrease in Denmark. However, long-term absences increased significantly in Denmark, while they decreased in Norway.

### Strengths and limitations

In this study, we used employee register data from two municipalities, which allowed us to perform a detailed comparison of sickness absence rates and sick leave episodes. The reliability of data from these registers is assumed to be high, and register data are considered a more objective measurement than, for example, self-reported data [1]. Errors may have occurred during data entry, which is impossible to check. We used individual-level data with complete and exact information on sick leave from day one, and harmonized the variables and definitions to make the dataset as precise as possible to ensure comparability. However, differences in definitions and registrations may cause challenges. Sickness absence during pregnancy seems to be recorded differently in Norway and Denmark. In Norway, sick leave among pregnant women is included in the general sickness absence statistics, while in Denmark it seems to be registered separately. It would have been preferable to have several years’ worth of information on sick leave for this time trend analysis, but we found significant changes over time in our study regardless.

Generally, register data are designed according to the criteria for sickness absence set by a country’s social security system, which can complicate between-country comparisons [11]. However, Gimeno [1] argued that international comparisons are needed to enable overall patterns to be observed, thereby indicating which policies are working from both a public health and economic standpoint. Unfortunately, for the health and care sector we lack international data for comparison of sickness absence patterns across countries.

Institutional arrangements, such as the design of employment protection, sick leave compensation scheme, and the transition from this scheme to other welfare schemes does have an impact on sickness absence levels [7,28]. In Norway, employees are strongly protected against losing their job, including when they are on sick leave, whereas employees in Denmark have weaker protection [29] and may lose their job during sickness absence. Employees with more sick leave episodes may be more likely to be fired in Denmark, and this may explain some of the difference in sickness absence rates in the two countries. More frequent short-term absence in Denmark may be due to the fear of losing the job if absent for longer. However, employment protection has not changed in recent years, and thus does not explain the convergence in sickness absence observed in the present study. Neither are there indications that the overall health of the employees in Norway and Denmark has changed over the years [2,3,30].

Westman and Etzion [31] studied the impact of vacation and job stress on burnout and absenteeism. They found a decline in absenteeism immediately after vacation and a return to pre-vacation levels four weeks later. This indicates that vacation might have a preventive effect on sickness absence. As sickness absence rates are lower in Denmark than in Norway, and the working year in Norway is two weeks longer than in Denmark, it could be hypothesized that longer vacation would protect against sickness absence. It may also be a matter of a ceiling effect, i.e., sickness absence in Norway is already so high that it is unlikely to become any higher, whereas in Denmark there is still room for an increase.

Previous studies have shown sickness absence to be negatively correlated with unemployment [20]. The disciplinary effects hypothesis may explain the relationship between sickness absence and unemployment [24]. In Norway, this correlation was strong until 2000, but it has been weak in recent years [24]. The unemployment rate is higher in Denmark than in Norway [5,6], which may explain some of the differences in sick leave rates between the two countries. However, the sickness absence rate increased and the unemployment rate decreased in Denmark from 2004 to 2008 [6,32]. Participation in the labor force is high in Norway and Denmark compared to other countries, especially when considering the participation of women and the elderly [2]; this may increase the sickness absence due to increased sickness absence rates in women and the elderly.

We found differences in sickness absence between age groups in both countries; however, the age distribution of sickness absence across countries was similar. Our findings are supported by a review of more than 185 studies that reported consistent age-related differences for a number of work attitudes and behaviors [23]. The lower sickness absence rates in older employees might be due to a healthy worker effect, e.g., the healthiest workers stay at work, and sicker employees may be selected to disability pensions or be otherwise out of work.

The observed increase in sickness absence rates and in sick leave episodes in young employees is serious. In a sickness absence trend study from England the highest absence rates were also found in young employees [33]. In a longitudinal study from 2004 to 2008 in Denmark, Pedersen [34] found that those aged 20–29 years had an increased risk of transition from work to sickness absence, and from sickness absence to unemployment. Both Norway and Denmark have difficulties recruiting youths to educational programs that lead to employment in the health and care sectors [35]. Gjerustad [36] found that achieving occupational aspiration was significantly related to sickness absence in a longitudinal study of Norwegian youths; a high occupational achievement indicated lower sickness absence. One could hypothesize that, if young employees choose their profession based not on their wishes but to avoid unemployment, their motivation could be low and their personal limits for reporting sick lowered [37]. In light of the large expansion of the working staff, such a hypothesis may partly explain the considerable increase in sickness absence among young employees observed in our study.

## Conclusions

We found an overall increasing trend in the sickness absence rate in Denmark, especially in the youngest age group. The number of sick leave episodes increased in both countries, but more so in Norway than in Denmark. In both countries, young employees had the largest increase in sickness absence rates and in sick leave episodes. In order to reduce sickness absence, preventive measures should target younger age groups. The results indicate that the two countries are converging in sickness absence measured as rate and episodes.

## Competing interests

The authors declare that they have no competing interests.

## Authors’ contributions

LK, TB, and RJ participated in the design of the study. NF and CMS provided data from Norway and Denmark, respectively. TB and LK did the data analysis. TB, RJ, CVN, NF, CJ, and CMS commented on the draft. LK was the main author of the manuscript. All authors read and approved the final manuscript.
